# The Impact of Test and Sample Characteristics on Model Selection and Classification Accuracy in the Multilevel Mixture IRT Model

**DOI:** 10.3389/fpsyg.2020.00197

**Published:** 2020-02-14

**Authors:** Sedat Sen, Allan S. Cohen

**Affiliations:** ^1^College of Education, Harran University, S̨anliurfa, Turkey; ^2^College of Education, University of Georgia, Athens, GA, United States

**Keywords:** item response theory, mixture item response model, multilevel data, model selection, classification accuracy

## Abstract

The standard item response theory (IRT) model assumption of a single homogenous population may be violated in real data. Mixture extensions of IRT models have been proposed to account for latent heterogeneous populations, but these models are not designed to handle multilevel data structures. Ignoring the multilevel structure is problematic as it results in lower-level units aggregated with higher-level units and yields less accurate results, because of dependencies in the data. Multilevel data structures cause such dependencies between levels but can be modeled in a straightforward way in multilevel mixture IRT models. An important step in the use of multilevel mixture IRT models is the fit of the model to the data. This fit is often determined based on relative fit indices. Previous research on mixture IRT models has shown that performances of these indices and classification accuracy of these models can be affected by several factors including percentage of class-variant items, number of items, magnitude and size of clusters, and mixing proportions of latent classes. As yet, no studies appear to have been reported examining these issues for multilevel extensions of mixture IRT models. The current study aims to investigate the effects of several features of the data on the accuracy of model selection and parameter recovery. Results are reported on a simulation study designed to examine the following features of the data: percentages of class-variant items (30, 60, and 90%), numbers of latent classes in the data (with from 1 to 3 latent classes at level 1 and 1 and 2 latent classes at level 2), numbers of items (10, 30, and 50), numbers of clusters (50 and 100), cluster size (10 and 50), and mixing proportions [equal (0.5 and 0.5) vs. non-equal (0.25 and 0.75)]. Simulation results indicated that multilevel mixture IRT models resulted in less accurate estimates when the number of clusters and the cluster size were small. In addition, mean Root mean square error (RMSE) values increased as the percentage of class-variant items increased and parameters were recovered more accurately under the 30% class-variant item conditions. Mixing proportion type (i.e., equal vs. unequal latent class sizes) and numbers of items (10, 30, and 50), however, did not show any clear pattern. Sample size dependent fit indices BIC, CAIC, and SABIC performed poorly for the smaller level-1 sample size. For the remaining conditions, the SABIC index performed better than other fit indices.

## Introduction

Item response theory (IRT; Lord and Novick, 1968) models have been used extensively for a variety of testing situations. However, traditional IRT models assume a single homogenous population which may be violated in some real data situations with multiple albeit latent subpopulations. Mixture extensions of IRT models have been proposed to account for heterogeneity due to these latent populations (Mislevy and Verhelst, 1990; Rost, 1990). Mixture IRT models combine a latent class model and an IRT model in a single model. Combining both models provides both qualitative and quantitative results simultaneously about the test and examinees by accounting for both categorical latent variables (i.e., latent classes) and continuous latent variables (i.e., factors) (e.g., Rost, 1990). Mixture IRT models have been used frequently due to their utility for measuring individual differences, when distinct subpopulations are present in the overall population (see Sen and Cohen, 2019, for a review of applications of mixture IRT models).

The single-level mixture IRT models are like multigroup item response models (Bock and Zimowski, 1997) in that groups are treated as manifest. Groups are taken as latent classes, however, in mixture IRT models. These models are useful for heterogeneous samples, although they do not account for the dependencies present in a multilevel (hierarchical) structure, such as are common in educational and psychological data. Ignoring the hierarchical structure with lower-level units aggregated in higher-level units has been shown to yield less accurate results because of violation of the local independence assumption (Lee et al., 2018). The hierarchical structure should be considered, in other words, in analyses of data from multilevel clusters. In this regard, multilevel mixture IRT models have been developed to account for possible dependencies, such as can arise due to cluster or multistage sampling (Vermunt, 2007). The dependency in multilevel data structures can be modeled in a straightforward way in a multilevel framework. These models can then be used to obtain information at both the individual (i.e., within) level and group (i.e., between) level. Students or examinees can be used to represent within-level and classrooms or schools can be used to represent between-level classes. Within-level latent classes capture the association between the responses at the student-level unit while between-level latent classes capture the association between the students within school-level units (Vermunt, 2003; Cho and Cohen, 2010).

As described in Lee et al. (2018), the two-parameter multilevel mixture item response model can be written as:

(1) $$\text{logit}\left[P\left(Y_{j\,k\,i}=1|\theta_{j\,k\,g},\theta_{k},C_{j\,k}\right)\right]=\alpha_{i\,g.W}\theta_{j\,k\,g}+\alpha_{i.B}\theta_{k}-\beta_{i\,g},$$

where *Y*_*jki*_ represents the responses of person *j* nested within the *k*th cluster (*k* = 1…,*K*) to item *i*, *C*_*jk*_ is a within-level latent classification variable where *Cj* = 1,., *g*,.,*G* for person *j* nested within cluster *k*, α_*ig.W*_ represents a within-level item discrimination parameter, α_*i.B *_represents between-level item discrimination parameter, β_*ig*_ is a class-specific item location parameter, θ_*jkg*_ is a class-specific within-level continuous latent variable $\sigma_{g}^{2}$ and θ_*k*_ represents a between-level continuous latent variable. Both θ_*jkg*_ and θ_*k*_ are assumed to follow normal distributions with a mean of zero and variance $\sigma_{g}^{2}\,\text{and}\,\tau^{2}$, respectively.

The multilevel mixture IRT models have interested researchers due to their utility for correctly accounting for dependencies among the data in multilevel data structures (Vermunt, 2008; Cho and Cohen, 2010; Tay et al., 2011; Bacci and Gnaldi, 2012, 2015; Varriale and Vermunt, 2012; Cho et al., 2013; Finch and Finch, 2013; Bennink et al., 2014; Jilke et al., 2015; Liu et al., 2018). Cho and Cohen (2010), Finch and Finch (2013), and Bennink et al. (2014) describe applications of different types of multilevel mixture IRT models for detection of differential item functioning (DIF). Bacci and Gnaldi (2012, 2015), and Vermunt (2008) analyzed educational data sets using multilevel mixture IRT models. Examples of other studies using multilevel mixture IRT models are analysis of self-reported emotions (Tay et al., 2011) and measurement non-equivalence (Jilke et al., 2015).

The exploratory use of multilevel mixture IRT modeling is based on the comparison of alternative models using relative fit indices such as the Akaike Information Criterion (AIC;Akaike, 1974) and Bayesian Information Criterion (BIC; Schwarz, 1978) indices. The successful applications of these models partly depend on selecting the correct model and its classification accuracy. Several studies have been conducted on model selection and classification accuracy issues with different mixture IRT models (Li et al., 2009; Preinerstorfer and Formann, 2012; Choi et al., 2017; Lee et al., 2018; Sen et al., 2019). Most of these studies focused on single-level mixture IRT models. Simulation studies conducted by Li et al. (2009) and Preinerstorfer and Formann (2012) suggested that BIC performed best among the model selection indices selected in dichotomous mixture IRT models. Similar results were reported by Sen et al. (2019) for multilevel mixture Rasch models. Lee et al. (2018) found BIC to better perform than AIC in selecting the correct multilevel model compared to a single level model. Previous studies on single level mixture IRT models reported that performances of model selection indices and the classification accuracy of these models can be affected by several factors including percentage of class-variant items, magnitude of item difficulty differences, pattern of item difficulty differences, mixing proportion of latent classes (Choi et al., 2017). Choi et al. (2017) found that AIC, corrected AIC (AICC; Sugiura, 1978), BIC, and sample-size adjusted BIC (SABIC; Sclove, 1987) performed differently depending on the percentage of class-variant items and the magnitude and pattern of item difficulty differences under a two-class structure. There appear to be no studies yet reported, however, examining these issues in multilevel extensions of mixture IRT models. Thus, the current study aims to investigate the effects of various class distinction features on the model selection, classification accuracy and quality of parameter recovery in multilevel mixture IRT models. The current study focused on the effects of class distinctive features on fitting a multilevel mixture 2-parameter logistic IRT model (Multilevel Mix2PL). Although the graded response model (GRM; Samejima, 1969) is common in psychological studies, the 2PLM essentially represents a simpler case of the GRM that; it was used as a starting point for investigating the research questions posed in the current study. To this end, this study investigated the following three research questions:

(1) How do the different test characteristics affect the quality of parameter estimates in multilevel mixture IRT models?
(2) How do these different characteristics affect classification accuracy in multilevel mixture IRT models?
(3) How do the model selection indices perform in the presence of these different characteristics?

## Materials and Methods

A Monte Carlo simulation study was conducted to answer the three research questions. Details of the simulation study are given below.

### Design of the Simulation Study

Data were simulated based on the dichotomous multilevel mixture IRT model (Lee et al., 2018) having two between-level and two within-level latent classes (labeled here as CB2C2). The generating parameters for the study were obtained from estimates of an empirical data set. Item threshold values obtained from this data set were used in data generation (see Supplementary Data Sheet S2). All data sets were generated with the Mplus 7.4 software package (Muthén and Muthén, 1998–2015) using the Mplus syntax provided by Lee et al. (2018) (see Supplementary Data Sheet S1). Different data sets were generated for a varying number of conditions using the MONTE CARLO simulation implemented in Mplus. The following conditions were simulated: number of items (10, 30, and 50), mixing proportions (equal and not equal), percentage of class variant items (30, 60, and 90%), number of clusters (50 and 100), and cluster size (10 and 50).

Ten-item test was used to represent a short test condition, a 30-item test was used to represent a medium test length and a 50-item test was used to represent a long test. Two different mixing proportions were included to investigate the effect of different mixing proportions, π: equal mixing proportions (π_1_ = π_2_ = 0.5) and unequal mixing proportions (π_1_ = 0.75, π_2_ = 0.25). Items with the same item threshold parameters across latent classes are considered class-invariant items, and items having unequal threshold parameters are considered class-variant items. Given that the number of class-variant items has been shown to affect number of detected latent class (Choi et al., 2017), different percentages of class-variant items were manipulated in this simulation study. The percentage of class-variant items manipulated in the simulation study was 30, 60, and 90% of items on the simulated tests. Number of clusters and cluster size have also been found to affect multilevel mixture IRT results (Lee et al., 2018). Thus, the numbers of clusters manipulated in the simulation study were 50 and 100 and the cluster sizes manipulated in the simulation study were 10 and 50. Overall, 72 conditions were simulated in this study (3 numbers of items × 2 mixing proportions × 3 class variant item percentages × 2 number of clusters × 2 cluster size). One hundred replications were generated for each condition.

### Estimation

Four different models were estimated: CB1C2, CB2C2, CB2C3 and CB3C3, CB is the notation for level-2 and C is the notation for level-1. Thus, CB1C2 represents a model with one level-two class and two level-one classes, CB2C2 represents a model with two level-one classes and two level-two classes, CB2C3 represents a model with level-two classes and three level-one classes, etc. The true (i.e., generating) model in this simulation study was the CB2C2 model, i.e., a multilevel mixture item response model with two within-level and two between-level latent classes. Thus, misspecified models were the CB1C2, CB2C3 and CB3C3 models. The total number of runs was 28,800 (=100 replications × 4 models × 72 conditions). Marginal maximum-likelihood estimation with the MLR estimator option was used as implemented in Mplus for estimation of the multilevel mixture IRT models. The following Mplus options were used: TYPE = TWOLEVEL MIXTURE; ALGORITHM = INTEGRATION; PROCESSORS = 2;. The Mplus syntax for model estimation is provided in the Supplementary Data Sheet S1.

### Evaluation Measures (RMSE-Model Selection)

#### Item Parameter Recovery Analysis

Root mean square error (RMSE) statistics were calculated, after item parameter estimates were placed onto the scale of the generating parameters, to examine the recovery of the generating parameters. RMSE was calculated between item threshold parameters of the true model and the estimated model using $\sqrt{\sum_{r=1}^{R}{\left({\hat{\lambda }}_{i}-\lambda \right)}^{2}/R,}$ where *r* represents the *r*th replication (*r* = 1,…,*R*).

Label switching can be a concern with mixture IRT estimation. Estimated latent classes can be switch across different replications. As an example, between-level latent class 2 on one data set can potentially correspond to between-level class 1 on another data set. Therefore, results for each data set were monitored to detect and, if necessary, to correct label switching. Threshold values obtained from the class were then used to appropriately calculate RMSE values.

### Classification Accuracy Rate

In the mixture IRT framework, each respondent has an estimated posterior probability for membership in each latent class. Each respondents is assigned to a single class based on their highest estimated posterior probability value. As described in Lee et al. (2018, p. 143), for each person *j* nested within cluster *k*, the posterior probability for membership in each latent class, *P*_*jkg*_, can be calculated as follows:

$$P_{j\,k\,g}=\frac{\begin{array}{c} {\hat{\pi }}_{g}.\prod_{i=1}^{I}{\left(P\left(y_{j\,k\,i}=1|{\tilde{\theta }}_{j\,k\,g},{\tilde{\theta }}_{k},C_{j\,k}\right)\right)}^{y_{j\,k\,i}}\\ {\left[1-P\left(y_{j\,k\,i}=1|{\tilde{\theta }}_{j\,k\,g},{\tilde{\theta }}_{k},C_{j\,k}\right)\right]}^{1-y_{j\,k\,i}} \end{array}}{\begin{array}{c} \sum_{g=1}^{G}{\hat{\pi }}_{g}.{\hat{\pi }}_{g}.\prod_{i=1}^{I}{\left(P\left(y_{j\,k\,i}=1|{\tilde{\theta }}_{j\,k\,g},{\tilde{\theta }}_{k},C_{j\,k}\right)\right)}^{y_{j\,k\,i}}\\ {\left[1-P\left(y_{j\,k\,i}=1|{\tilde{\theta }}_{j\,k\,g},{\tilde{\theta }}_{k},C_{j\,k}\right)\right]}^{1-y_{j\,k\,i}} \end{array}},$$

where *Y*_*jki*_ represents the responses of person *j* nested within *k*th cluster to item *i*, and *k* represents cluster *k* (*k* = 1,.,*K*), *C*_*jk*_ is a categorical latent variable at the within level, ${\hat{\pi }}_{g}$ is an estimated mixing proportion, ${\tilde{\theta }}_{j\,k\,g}$ is a class-specific within-level predicted score, and θ_*k*_ represents a between-level predicted score. The *P*_*jkg*_ values sum to 1 for each person ($\text{i}.\text{e}.,\sum_{g=1}^{G}P_{j\,k\,g}=1)$.

Simulated examinees were assigned to specified latent classes during data generation. It is necessary to determine whether these examinees were classified into the same latent classes after model estimation. Posterior probabilities for membership of each examinee were calculated using the CPROBABILITIES option of the SAVEDATA command in Mplus. Classification accuracy rate was calculated for each condition. The correct detection rate was defined as the correct classification of the latent class membership for each examinee. Generated and simulated class memberships were compared and a percentage was computed across the 100 replications for each condition. Thus, agreement was recorded when an examinee assigned to the first class (Class 1) during data generation was also classified into Class 1 after estimation.

#### Model Selection

Unlike multigroup IRT models, the latent classes in mixture IRT models are not known *a priori* in an exploratory analysis as they are unobserved. In an exploratory analysis, different numbers of latent classes are specified as candidate models and estimated for a given data set. The most commonly used criteria for model selection in IRT models are based on either a likelihood ratio test or information criterion indices. Nylund et al. (2007) note that the likelihood ratio test is not appropriate for model selection for mixture IRT models. Thus, information criterion indices were used for model selection in this study.

Information criterion indices are based on some form of penalization of the loglikelihood. The penalization is used to adjust for the selection of over-parameterized models. Let *L* be the likelihood function obtained from maximum likelihood estimation and *P* be the penalty term. The following is a general form for information criterion indices:

-2⁢log⁢L+P

The performances of AIC, BIC, consistent AIC (CAIC; Bozdogan, 1987), and SABIC were investigated in this study as these are generally the more commonly used indices in mixture IRT applications (Sen and Cohen, 2019). Each of these indices applies a different penalty function to the −2log*L* term. Thus, the definitions of the relative fit indices in this study are as follows:

AIC=-2⁢log⁢L+2⁢d,

BIC=-2⁢log⁢L+d.ln⁢(N),

CAIC=-2⁢log⁢L+d.[ln⁡(N)+1],

SABIC=-2⁢log⁢L+d.ln⁢[(N+2)/24],

Where, *N* represents the number of examinees and *d* represents the number of parameters. Smaller numbers for these fit indices indicate better fit. Performances of these indices were examined by calculating the proportion of correct selections for each model. To evaluate correct model selections, the data sets generated based on CB2C2 model were analyzed with four different models (i.e., CB1C2, CB2C2, CB2C3, and CB3C3). The correct detection rate was defined as the correct detection of the simulated CB2C2 model with the correct number of within- and between-level latent classes.

## Results

### Parameter Recovery

Table 1 presents mean RMSE values for each condition. The labels indicate the condition under which the data were generated. For example, the label E5010 indicates that the CB2C2 data were generated for equal mixing proportions for 50 clusters and with a cluster size of 10. That is, number of level-2 units is 50 and number of level-1 is 10. The NE label indicates unequal mixing proportion conditions. Results of each condition are presented for 10-, 30-, and 50-item test lengths and 30, 60, and 90% of class variant items. Mean RMSE values for item threshold estimates ranged from 0.092 to 2.927.

**TABLE 1:** Mean RMSE values of item threshold estimates for the CB2C2 Model.

	**Percent of class variant items**								
**Simulation condition**	**10 Items**			**30 Items**			**50 Items**		
	**30**	**60**	**90**	**30**	**60**	**90**	**30**	**60**	**90**
E5010	1.335	1.812	1.949	0.454	0.993	1.333	0.562	1.231	2.007
E5050	0.256	0.325	0.829	0.118	0.732	0.977	0.107	0.985	1.268
E10010	0.752	0.830	1.099	0.213	0.766	1.007	0.199	1.006	1.458
E10050	0.164	0.191	0.767	0.083	0.724	0.965	0.075	0.977	1.260
NE5010	1.087	1.213	1.401	1.873	2.653	2.710	1.435	1.860	2.927
NE5050	0.400	0.596	1.010	0.328	0.751	1.087	0.134	0.988	1.321
NE10010	0.803	1.377	1.565	1.289	1.621	1.928	0.548	1.120	1.766
NE10050	0.335	0.376	0.859	0.328	0.734	1.070	0.092	0.979	1.262

*E, Equal proportion; NE, Non-equal proportions; E5010 reprents a condition with equal mixing proportions under 50 clusters and with a cluster size of 10.*

As shown in Table 1, the mean RMSE values decreased as the cluster size and number of examinees for level-1 increased. Similarly, mean RMSE values increased as the percentage of class-variant items increased. As expected, greater accuracy was observed with the higher number of clusters and cluster size conditions. Type of mixing proportion (equal vs. unequal) and number of items (10, 30, and 50) did not show any clear pattern of recovery.

### Classification Accuracy

As with latent class models, mixture IRT models assign each examinee to one of the latent classes based on class probability values. The class memberships created during the data generation were compared with the estimated class memberships. A classification accuracy rate was calculated for each condition between generated values and estimated values based on the same model. Classification accuracy rates are shown in Table 2. These rates ranged from 12.31 to 90.64%. Table 2 shows that the classification accuracy rates increase as the number of items increases. The highest rates occurred for the 50-item conditions while the lowest rates were observed with 10-item conditions. Only the 30-item conditions with 60% of class-variant items did not follow this pattern. This condition actually yielded lower rates than the 10-item counterparts (i.e., 10-item conditions with 60% of class variant items). Equal mixing proportion conditions yielded smaller accuracy rates than unequal mixing proportion conditions for almost each percentage of class-variant items and test length conditions. As shown in Table 2, conditions with 60% of class-variant items yielded higher accuracy rates than conditions with 30 and 90% of class-variant items under 10- and 50-item condition. However, this was not the case with the 30-item conditions. The cluster size seemed to influence the classification accuracy rates. The conditions with the smaller level-1 sample size (i.e., 10) yielded lower accuracy rates than the conditions with the higher level-1 sample size (i.e., 50). Similarly, the number of clusters appeared to influence classification accuracy rates. The conditions with 50 clusters yielded lower accuracy rates than the conditions with 100 clusters. As expected, increases in the number of items, number of clusters and cluster size had a positive effect on classification accuracy.

**TABLE 2:** Classification accuracy rates for CB2C2 Model.

**Simulation condition**	**10 Items**			**30 Items**			**50 Items**		
	**30**	**60**	**90**	**30**	**60**	**90**	**30**	**60**	**90**
E5010	37.35	38.20	31.43	43.19	24.14	44.66	69.11	80.04	69.38
E5050	45.13	58.58	38.69	57.86	45.05	38.85	82.29	89.02	86.92
E10010	30.82	42.54	27.87	44.18	27.58	58.00	70.04	83.34	78.93
E10050	35.39	61.53	30.18	61.15	47.43	37.79	82.09	89.02	87.12
NE5010	37.00	37.42	30.93	28.50	27.27	26.69	65.86	74.70	45.70
NE5050	52.94	57.05	45.03	38.71	26.58	29.01	85.04	90.50	88.61
NE10010	34.79	47.14	36.97	26.61	32.31	32.50	72.86	85.12	66.97
NE10050	60.27	57.42	32.45	31.13	12.31	15.87	85.85	90.64	86.52

*E, Equal proportion; NE, Non-equal proportions; E5010 reprents a condition with equal mixing proportions under 50 clusters and with a cluster size of 10.*

### Model Selection

AIC, BIC, CAIC, and SABIC values were calculated for each condition. The number of correct selections was calculated as the number of detections of the CB2C2 (i.e., the generating) model over 100 iterations. The frequencies of correct model selections are shown in Tables 3–5 for each of the information indices.

**TABLE 3:** Number of correct detections over 100 replications for 10-Item conditions.

	**AIC**			**BIC**			**SABIC**			**CAIC**		
	**30**	**60**	**90**	**30**	**60**	**90**	**30**	**60**	**90**	**30**	**60**	**90**
E5010	82	52	65	3	0	2	59	31	48	2	0	0
E5050	82	76	97	100	100	100	100	100	100	97	99	98
E10010	86	67	67	21	0	3	84	58	65	7	0	1
E10050	57	70	89	80	100	100	77	100	100	77	97	97
NE5010	70	57	69	1	0	2	51	26	41	0	0	0
NE5050	91	79	90	100	80	100	100	87	100	97	77	95
NE10010	86	74	73	11	1	2	78	42	70	5	0	2
NE10050	75	38	92	100	70	100	100	59	100	100	73	97

*E, Equal proportion; NE, Non-equal proportions; E5010 reprents a condition with equal mixing proportions under 50 clusters and with a cluster size of 10; AIC, Akaike Information Criterion; BIC, Bayesian Information Criterion; CAIC, Consistent AIC; SABIC, Sample size adjusted BIC.*

**TABLE 4:** Number of correct detections over 100 replications for 30-Item conditions.

	**AIC**			**BIC**			**SABIC**			**CAIC**		
	**30**	**60**	**90**	**30**	**60**	**90**	**30**	**60**	**90**	**30**	**60**	**90**
E5010	53	55	47	28	0	0	99	97	66	11	0	0
E5050	56	72	37	100	100	100	100	100	100	100	100	100
E10010	48	34	48	99	53	0	100	99	66	97	20	0
E10050	59	77	41	99	100	100	99	100	100	98	100	100
NE5010	28	38	25	0	2	0	11	6	100	0	0	0
NE5050	18	65	53	81	66	8	97	99	83	66	33	1
NE10010	16	47	31	0	0	0	13	6	1	0	0	0
NE10050	5	63	39	100	99	92	85	99	99	100	98	85

*E, Equal proportion; NE, Non-equal proportions; E5010 reprents a condition with equal mixing proportions under 50 clusters and with a cluster size of 10; AIC, Akaike Information Criterion; BIC, Bayesian Information Criterion; CAIC, Consistent AIC; SABIC, Sample size adjusted BIC.*

**TABLE 5:** Number of correct detections over 100 replications for 50-Item conditions.

	**AIC**			**BIC**			**SABIC**			**CAIC**		
	**30**	**60**	**90**	**30**	**60**	**90**	**30**	**60**	**90**	**30**	**60**	**90**
E5010	58	79	78	0	0	1	54	30	2	0	0	0
E5050	67	66	77	100	100	100	100	100	100	100	100	90
E10010	67	76	92	1	0	0	93	89	21	0	0	0
E10050	69	65	65	100	100	97	100	100	94	100	100	98
NE5010	57	49	31	0	0	0	23	3	0	0	0	0
NE5050	77	74	76	100	89	36	100	99	100	99	78	12
NE10010	60	73	97	0	0	0	53	26	0	0	0	0
NE10050	92	91	68	100	100	100	100	100	100	100	100	98

*E, Equal proportion; NE, Non-equal proportions; E5010 reprents a condition with equal mixing proportions under 50 clusters and with a cluster size of 10; AIC, Akaike Information Criterion; BIC, Bayesian Information Criterion; CAIC, Consistent AIC; SABIC, Sample size adjusted BIC.*

The numbers of correct detections for 10-item conditions are presented in Table 3. Correct detection frequencies ranged between 0 and 100 out of 100 replications in the 10-item conditions. As shown in Table 3, BIC, CAIC, and SABIC performed better than AIC index for the conditions with level-1 sample size of 50 (i.e., E5050, E10050, NE5050, and NE10050). The number of correct detections of the BIC and CAIC indices for the smaller number of level-1 sample size conditions were all either very low or zero except for unequal mixing proportion condition with 100 clusters and level-1 sample size of 10 (i.e., NE10010). The SABIC index performed better than BIC index for almost all conditions. BIC and CAIC performed less well than the SABIC for the small level-1 sample. However, the level-1 sample size did not appear to have any effect on the performance of AIC. The percentage of class-variant items appeared to influence the correct detection rates based on four fit indices. The 60% conditions yielded lower correct detection rates for almost every condition. The effects of mixing proportion type (equal vs. unequal), however, did not show any clear pattern.

The number of correct detections for the 30-item conditions ranged between 0 and 100 (see Table 4). As shown in Table 4, BIC, CAIC, and SABIC performed better than AIC for the sample size of 50 (i.e., E5050, E10050, NE5050, and NE10050). As was the case for the 10-item conditions, the numbers of correct detections of the BIC and CAIC indices for smaller number of level-1 sample size conditions were all either very low or zero for the E5010 and E10010 conditions. SABIC performed better than BIC and CAIC for most conditions except for NE10050 condition under 30% of class-variant items. The small level-1 sample size (i.e., 10) appeared to influence the performance of BIC and CAIC compared to SABIC. However, the level-1 sample size did not show any clear pattern for the performance of AIC. The percentage of class-variant items appears to influence the correct detection rates based on four fit indices. The 60% conditions yielded lower correct detection rates for most of the conditions. The effects of mixing proportion type (equal vs. unequal), however, did not show any clear pattern.

Correct detection frequencies (see Table 5) ranged between 0 and 100 in the 50-item conditions. As shown in Table 5, BIC, CAIC, and SABIC performed better than AIC for the conditions with the level-1 sample size of 50 (i.e., E5050, E10050, NE5050, and NE10050). AIC performed better than BIC, CAIC, and SABIC, however, for the conditions with the level-1 sample size of 10 (i.e., E5010, E10010, NE5010, and NE10010). As was the case with the 10- and 30-item conditions, the numbers of correct detections of the BIC and CAIC indices for smaller level-1 sample size conditions were all either very low or zero for the 50-item conditions. SABIC performed better than BIC and CAIC for most conditions except for E10050 for the 90% class-variant items condition. The small level-1 sample size (i.e., 10) appears to influence the performance of BIC and CAIC compared to SABIC. The level-1 sample size, however, did not show any clear pattern for AIC. Similarly, the percentage of class-variant items and the effects of type of mixing proportion (i.e., equal vs. unequal) did not show any clear pattern.

## Summary and Discussion

This simulation study examined the accuracy of parameter estimates and classifications under different multilevel and mixture conditions. The simulation factors in this research were chosen to represent different class-distinction features in multilevel mixture IRT modeling, in which the percentage of class-variant items, the number and magnitude of clusters, and the number of items varied for the structure with two level-1 and two level-2 classes (i.e., CB2C2 model). In addition, this study also investigated the differential performance of the four information criteria (AIC, BIC, CAIC, and SABIC) for model selection with different multilevel mixture IRT model applications.

Findings from the simulation study indicated that greater accuracy was observed with the higher number of clusters (i.e., 100 clusters) and cluster size (i.e., 50 simulated examinees) conditions, as well as the lower (30%) percentage of class-variant item conditions. When the number of clusters and the cluster sizes were small, the applications of multilevel mixture IRT models can be problematic with respect to the accuracy of item parameter estimates. These findings were consistent with previous research by Lee et al. (2018) which found that the multilevel mixture IRT model does not perform well for small sample sizes.

Findings regarding classification accuracy rates showed that the classification accuracy rates increased as the number of items increased. Equal mixing proportion conditions yielded smaller accuracy rates than unequal mixing proportion conditions for most percentages of class-variant items and test length conditions. The numbers of clusters and cluster size appeared to influence classification accuracy rates. The smaller cluster size (i.e., 10 examinees) and smaller number of clusters (i.e., 50 clusters) yielded lower accuracy rates. Similarly, the number of clusters appeared to influence classification accuracy rates. As expected, increases in the number of items, number of clusters and cluster size had a positive effect on classification accuracy.

Differential performances of the AIC, BIC, CAIC, and SABIC were observed under the different study conditions. Overall, SABIC performed better than BIC or CAIC for the small level-1 sample (i.e., 10) conditions, and for the conditions with the higher sample size at level-1 (i.e., 50). BIC and CAIC failed to select the true model for conditions with the smaller level-1 sample size. Overall, BIC and CAIC indices showed similar performances under the different data conditions. The SABIC appears to be the better than BIC and CAIC for the smaller level-1 sample size. These findings were consistent with Choi et al. (2017) that showed the superiority of SABIC over other relative fit indices. AIC also appeared to perform better than SABIC, however, under some conditions (i.e., NE5010, NE10010 conditions with 10-, 30- and 50-items and E5010, E10010 conditions with 10- and 50-items). Thus, results suggest that no uniformly superior single information criterion index of the four indices studied here was consistently the best model selection index under each of the simulated conditions here.

Multilevel mixture IRT models and relative fit indices used for model selection perform better with higher number of clusters and cluster sizes. The percentage of class-variant items also appeared to have an effect on accuracy of model estimates and on performance of model selection indices. Given these findings, it is important to note that model selection also needs to pay attention to substantive theory as well as to multiple fit indices rather than relying on a single fit index for model selection. The present study shares similar limitations to those of other simulation studies using similar conditions in the study design (e.g., Choi et al., 2017; Lee et al., 2018).

## Data Availability Statement

Datasets generated for E5010 conditions of this study are included in the article/Supplementary Material.

## Author Contributions

Both authors contributed equally to the data analyses and reporting parts.

## Conflict of Interest

The authors declare that the research was conducted in the absence of any commercial or financial relationships that could be construed as a potential conflict of interest.
