# Supplementary material for: The Impact of Test and Sample Characteristics on Model Selection and Classification Accuracy in the Multilevel Mixture IRT Model
Source: Front Psychol. 2020 Feb 14;11:197. doi: 10.3389/fpsyg.2020.00197 (PMC7033749; doi:10.3389/fpsyg.2020.00197)
Supplement: Supplementary file 2 [file Data_Sheet_2.PDF]

Appendix

Table1. Item threshold values for generating CB2C2 Model with 10 items

Item	CB1C1	CB1C2	CB2C1	CB2C2
1	**-2.831**	**-0.338**	**-1.520**	**0.937**
2	**-2.531**	**-1.101**	**-1.247**	**0.590**
3	**-0.690**	**1.120**	**0.378**	**2.058**
4	-0.517	-0.517	-0.517	-0.517
5	-0.110	-0.110	-0.110	-0.110
6	1.725	1.725	1.725	1.725
7	-2.666	-2.666	-2.666	-2.666
8	2.822	2.822	2.822	2.822
9	2.861	2.861	2.861	2.861
10	1.813	1.813	1.813	1.813

Note. 30% of items are class-variant items. The percentage of class-variant items were taken as 60%, and 90% of items by allowing 60% and 90% of the items to be different. CB1C1=Level-1 Class 1 within Level-2 Class 1; CB1C2=Level-1 Class 2 within Level-2 Class 1; CB2C1=Level-1 Class 1 within Level-2 Class 2; CB2C2=Level-1 Class 2 within Level-2 Class 2.

Table2. Item threshold values for generating CB2C2 Model with 30 items

Item	CB1C1	CB1C2	CB2C1	CB2C2
1	**0.191**	**3.428**	**2.636**	**2.345**
2	**-1.572**	**2.062**	**3.048**	**1.471**
3	**-0.932**	**1.281**	**2.233**	**0.832**
4	**-0.439**	**1.854**	**3.085**	**1.003**
5	**-1.788**	**0.422**	**2.164**	**0.282**
6	**-2.783**	**1.183**	**2.860**	**0.742**
7	**-0.762**	**0.530**	**1.288**	**0.494**
8	**-1.532**	**-0.132**	**0.374**	**-0.183**
9	**-1.407**	**0.274**	**1.461**	**-0.055**
10	-2.156	-2.156	-2.156	-2.156
11	3.335	3.335	3.335	3.335
12	-2.527	-2.527	-2.527	-2.527
13	-1.999	-1.999	-1.999	-1.999
14	-0.116	-0.116	-0.116	-0.116
15	-0.950	-0.950	-0.950	-0.950
16	-0.818	-0.818	-0.818	-0.818
17	-0.369	-0.369	-0.369	-0.369
18	-1.174	-1.174	-1.174	-1.174
19	-3.309	-3.309	-3.309	-3.309
20	0.362	0.362	0.362	0.362
21	-0.667	-0.667	-0.667	-0.667
22	0.795	0.795	0.795	0.795
23	-0.601	-0.601	-0.601	-0.601
24	1.763	1.763	1.763	1.763
25	-0.057	-0.057	-0.057	-0.057
26	-0.802	-0.802	-0.802	-0.802
27	-0.311	-0.311	-0.311	-0.311
28	-0.245	-0.245	-0.245	-0.245
29	0.343	0.343	0.343	0.343
30	-0.974	-0.974	-0.974	-0.974

Note. 30% of items are class-variant items. The percentage of class-variant items were taken as 60%, and 90% of items by allowing 60% and 90% of the items to be different. CB1C1=Level-1 Class 1 within Level-2 Class 1; CB1C2=Level-1 Class 2 within Level-2 Class 1; CB2C1=Level-1 Class 1 within Level-2 Class 2; CB2C2=Level-1 Class 2 within Level-2 Class 2.

Table3. Item threshold values for generating CB2C2 Model with 50 items

Item	CB1C1	CB1C2	CB2C1	CB2C2
1	**-2.194**	**-0.675**	**-0.97**	**0.613**
2	**-2.348**	**-1.061**	**-0.952**	**0.457**
3	**-0.657**	**1.416**	**0.384**	**2.145**
4	**-0.011**	**1.390**	**1.136**	**2.035**
5	**-0.078**	**0.46**	**0.653**	**0.774**
6	**1.726**	**2.901**	**2.925**	**2.269**
7	**-2.458**	**-0.488**	**-1.31**	**-0.061**
8	**3.573**	**3.734**	**2.266**	**2.535**
9	**-0.669**	**1.644**	**0.467**	**2.220**
10	**-0.091**	**1.795**	**0.832**	**1.769**
11	**-1.426**	**0.9**	**-0.152**	**1.413**
12	**0.502**	**2.495**	**1.633**	**2.998**
13	**-0.543**	**0.928**	**0.143**	**1.554**
14	**0.997**	**2.075**	**1.311**	**3.584**
15	**-2.348**	**-0.579**	**-1.182**	**0.231**
16	-1.049	-1.049	-1.049	-1.049
17	-0.825	-0.825	-0.825	-0.825
18	1.651	1.651	1.651	1.651
19	-1.339	-1.339	-1.339	-1.339
20	-1.254	-1.254	-1.254	-1.254
21	1.716	1.716	1.716	1.716
22	-0.100	-0.100	-0.100	-0.100
23	-0.159	-0.159	-0.159	-0.159
24	0.544	0.544	0.544	0.544
25	-1.283	-1.283	-1.283	-1.283
26	-1.114	-1.114	-1.114	-1.114
27	-0.389	-0.389	-0.389	-0.389
28	-1.131	-1.131	-1.131	-1.131
29	-0.986	-0.986	-0.986	-0.986
30	-1.256	-1.256	-1.256	-1.256
31	2.686	2.686	2.686	2.686
32	-2.670	-2.670	-2.670	-2.670
33	-1.809	-1.809	-1.809	-1.809
34	-0.296	-0.296	-0.296	-0.296
35	-1.075	-1.075	-1.075	-1.075
36	-0.345	-0.345	-0.345	-0.345
37	-0.310	-0.310	-0.310	-0.310
38	-0.844	-0.844	-0.844	-0.844
39	-3.803	-3.803	-3.803	-3.803
40	0.072	0.072	0.072	0.072
41	-0.146	-0.146	-0.146	-0.146
42	1.023	1.023	1.023	1.023

43	-1.045	-1.045	-1.045	-1.045
44	0.824	0.824	0.824	0.824
45	0.336	0.336	0.336	0.336
46	-1.018	-1.018	-1.018	-1.018
47	-0.765	-0.765	-0.765	-0.765
48	-0.096	-0.096	-0.096	-0.096
49	0.235	0.235	0.235	0.235
50	-0.990	-0.990	-0.990	-0.990

Note. 30% of items are class-variant items. The percentage of class-variant items were taken as 60%, and 90% of items by allowing 60% and 90% of the items to be different. CB1C1=Level-1 Class 1 within Level-2 Class 1; CB1C2=Level-1 Class 2 within Level-2 Class 1; CB2C1=Level-1 Class 1 within Level-2 Class 2; CB2C2=Level-1 Class 2 within Level-2 Class 2.
